# Poor sleep is associated with higher blood pressure and uterine artery pulsatility index in pregnancy: a prospective cohort study

**DOI:** 10.1111/1471-0528.16591

**Published:** 2020-11-23

**Authors:** Y Tang, J Zhang, F Dai, NS Razali, S Tagore, BSM Chern, KH Tan

**Affiliations:** ^1^ Department of Obstetrics and Gynaecology KK Women’s and Children’s Hospital Singapore City Singapore; ^2^ Ministry of Education – Shanghai Key Laboratory of Children’s Environmental Health Xinhua Hospital Shanghai Jiao Tong University School of Medicine Shanghai China; ^3^ Department of Maternal Fetal Medicine KK Women’s and Children’s Hospital Singapore City Singapore; ^4^ Department of Minimally Invasive Surgery KK Women’s and Children’s Hospital Singapore City Singapore

**Keywords:** Blood pressure, pregnancy, sleep quality, uterine artery Doppler

## Abstract

**Objective:**

To elucidate the association between sleep disturbances and blood pressure as well as uterine artery Doppler during pregnancy in women with no pre‐existing hypertension.

**Design:**

Prospective cohort study.

**Setting:**

Outpatient specialist clinics at KK Women’s and Children’s Hospital, Singapore.

**Population:**

Women with viable singleton pregnancies confirmed by ultrasonography at less than 14 weeks of amenorrhoea at first visit.

**Methods:**

In all, 926 subjects were recruited for this study in the outpatient specialist clinics at KK Women’s and Children’s Hospital, Singapore, between 1 September 2010 and 31 August 2014. They were followed up throughout pregnancy with sleep quality, blood pressure and uterine artery Doppler assessed at each visit.

**Main outcome measures:**

Sleep quality, blood pressure and uterine artery Doppler.

**Results:**

Sleep progressively worsened as pregnancy advanced. Shorter sleep duration and poorer sleep efficiency were associated with higher blood pressure, especially in the first trimester. Mixed model analysis demonstrated an overall positive association between sleep quality represented by Pittsburgh Sleep Quality Index (PSQI) score and diastolic blood pressure (DBP) (*P* < 0.001) and mean arterial pressure (MAP) (*P* = 0.005) during pregnancy after considering all trimesters. Sleep duration was found to be negatively associated with both systolic blood pressure (SBP) (*P* = 0.029) and DBP (*P* = 0.002), whereas sleep efficiency was negatively correlated with DBP (*P* = 0.002) only. Overall poor sleep during pregnancy was also found to be associated with a higher uterine artery pulsatility index.

**Conclusion:**

Our prospective study demonstrated that poor sleep quality is significantly associated with higher blood pressure and uterine artery pulsatility index during pregnancy.

**Tweetable abstract:**

Poor sleep quality is significantly associated with higher blood pressure and higher uterine artery pulsatility index during pregnancy.

## Tweetable abstract

Poor sleep quality is significantly associated with higher blood pressure and higher uterine artery pulsatility index during pregnancy.

## Introduction

The association between sleep disturbances and elevated blood pressure has been well studied.^1^, ^2^, ^3^, ^4^ However, relatively few studies have focused on the pregnant population. Sleep disturbances are very common throughout pregnancy^5^, ^6^ and can lead to significant adverse outcomes such as pregnancy‐induced hypertension, gestational diabetes, pre‐eclampsia and adverse cardiac events.^7^, ^8^, ^9^, ^10^, ^11^, ^12^, ^13^ Blood pressure varies throughout pregnancy. A cohort study by Grindheim et al.^14^ showed that both systemic blood pressure (SBP) and diastolic blood pressure (DBP) normally fall in early pregnancy, reaching a mean of 105/60 mmHg in the second trimester, and then gradually riseing to pre‐pregnancy values at term. William et al.^15^ showed a similar pattern. One prospective study using 24‐hour ambulatory blood pressure monitoring for four stages of pregnancy showed a gradual increase in BP as pregnancy advanced.^16^ The majority of previous studies investigating the effect of sleep on BP during pregnancy focused on the effect of sleep disturbances on the risk of pregnancy‐induced hypertension. One small study with 89 participants by Okada et al.^17^ showed that poor sleep in early pregnancy is associated with an increase in SBP from the first to third trimester. Thus far, there is no large cohort study focusing on sleep quality throughout pregnancy along with blood pressure changes among the pregnant population.

Uterine artery Doppler studies have long been employed to identify patients at risk of developing hypertensive disorders in pregnancy, especially pre‐eclampsia.^18^, ^19^ Resistance to blood flow in the uteroplacental circulation is reflected by increased uterine artery impedance, measured as pulsatility index (PI) and resistance index (RI).^20^ Uterine artery PI and RI values decrease with increasing gestational age as a result of trophoblastic invasion in a normal pregnancy.^20^ Derangement in trophoblastic differentiation hinders the physiological drop in uterine artery impedance during pregnancy and can potentially lead to hypertensive disorder and its associated complications, including fetal growth restriction and placenta abruption.^21^ Uterine artery impedance can be also affected by multiple factors such as hormonal changes, chronic androgenism in polycystic ovarian syndrome (PCOS) and antihypertensive use.^20^ The effect of sleep on uterine artery Doppler measures has been less well studied in the literature. One recent investigation found no association between Epworth Sleepiness Scale score and uterine artery PI.^22^

In this prospective study, we aim to delineate the association between sleep quality and BP throughout the entire pregnancy in individuals with no pre‐existing hypertension. Uterine artery PI and RI were also assessed in relation to sleep quality.

## Materials and methods

### Study population

The Neonatal and Obstetric Risk Assessment (NORA) study is a longitudinal prospective study conducted in KK Women’s and Children’s Hospital, Singapore, between 1 September 2010 and 31 August 2014. Participants were women with singleton pregnancies who attended antenatal clinics from their first trimester. Women with viable singleton pregnancies confirmed by ultrasonography at less than 14 weeks of amenorrhoea were considered eligible. Patients with chronic medical conditions known to be associated with adverse pregnancy outcomes (such as systemic lupus erythematosus) and pregnancies complicated by aneuploidy or fetal anomalies were excluded. Women with unsuccessful pregnancies (termination, miscarriage) were also excluded. Enrolment details have been described elsewhere.^23^ Briefly, 3271 women were identified, of whom 1013 consented to participate in the study. A total of 934 women (92%) completed all four antenatal follow‐up visits and 926 women had complete delivery information. Gestational age was calculated from the estimated due date, which was confirmed from the ultrasound scan in the first trimester based on crown−rump length. In the present analysis, 10 women who had pre‐existing hypertension were excluded, bringing the final number of subjects to 916. A written informed consent was obtained from all participants.

### Covariates

Information on socio‐demographic and lifestyle characteristics was collected mainly during the first antenatal visit: age, parity, race, education level, marital status, occupation, income, BMI, lifestyle characteristics such as drinking, smoking, exercise and caffeine intake. BMI and lifestyle characteristics were also recorded at all subsequent visits. Age and BMI were continuous variables and all the other factors were categorical variables.

Sleep quality was assessed via the Pittsburgh Sleep Quality Index (PSQI) questionnaire during four visits: 9–14 weeks of gestation (visit 1), 18–22 weeks of gestation (visit 2), 28–32 weeks of gestation (visit 3) and 34–39 weeks of gestation (visit 4). Four main questions were asked in the questionnaire:

(1) During the past month, what time have you usually gone to bed at night?

(2) During the past month, how long (in minutes) has it usually taken you to fall asleep each night?

(3) During the past month, what time have you usually got up in the morning?

(4) During the past month, how many hours of actual sleep did you get at night, which may be different from the number of hours you spent in bed?

PSQI score was calculated after combining the scores for each question. Women with poorer sleep quality have higher PSQI scores. Furthermore, specific aspects of sleep quality including sleep latency (question 2), sleep duration (question 4) and sleep efficiency (calculated as actual hours of sleep over total hours from time in bed to time of getting up) were individually assessed and compared in relation to blood pressure.

### Outcome

Non‐invasive systolic and diastolic blood pressure was taken during each visit using validated automated devices. Participants were allowed to rest for 5 minutes before trained nurses took their SBP and DBP. Three measurements were taken at 1‐minute intervals and the average was calculated for statistical analysis. MAP was calculated from SBP and DBP using the formula MAP = 1/3 SBP + 2/3 DBP. Bilateral uterine artery flow velocity waveforms with colour‐pulsed Doppler were performed during each visit via transabdominal ultrasound using GE Volusion (GE Healthcare, Singapore) by four trained sonographers. The sonographers reviewed their techniques and performances regularly to ensure consistency and quality of scan. Uterine artery was identified on the sagittal section of the uterus along the side of cervix and uterus at the level of internal os during the first visit.^24^ At subsequent visits, uterine artery was identified at the crossover with the external iliac artery.^20^ Pulsed wave Doppler was used with the sampling gate set at 2 mm. Pulsatility and resistance indexes were measured after three similar waveforms were obtained. The mean of bilateral PI and UI was calculated for statistical analysis.

### Statistical analysis

Statistical analysis of the data was performed using SPSS 22.0 (IBM, Armonk, NY, USA). Normality of continuous variables was assessed by the Kolmogorov−Smirnov test combined with P‐P plots. Normally distributed data were expressed as mean ± standard deviation and non‐normally distributed data as median (interquartile range). Categorical variables were described as the sample number and frequency of each category. Stepwise linear regression was performed across the four visits on the following variables: PSQI, sleep latency, sleep duration and sleep efficiency, to establish the association between sleep quality and blood pressure throughout pregnancy, after considering potential confounding factors. Data from each individual visit were analysed separately. Mixed model analysis was employed to study the overall association between sleep and BP as well as sleep and uterine artery resistance and pulsatility index, combining the information from all four antenatal visits. Analysis was adjusted for possible confounders. All the 916 subjects were included in the analysis (including the 38 patients who developed pregnancy‐induced hypertension or pre‐eclampsia [4.3% of all participants])^23^.

The NORA study was approved by the SingHealth Centralised Institutional Review Board Ethics Committee, Singapore (CIRB Ref No. 2010/214/D). There was no public or patient involvement in the study design or interpretation of results. Singapore National Medical Research Council (NMRC) Collaborative Centre Grant (IPRAMHO) supported this study.

## Results

As shown in Table 1, our study subjects consist of a multiracial population with an average age of 30 years and different educational levels, occupations and social status. Chinese and Malay women made up most of our study subjects (50.7 and 27.3%, respectively) with small numbers of Indians and others. Most of the subjects have education levels of high school/junior college and above. Their household income ranges from <3500 to more than 8500 Singapore dollars a month, with most women working as white‐collar workers. A similar percentage of nulliparous (54.4%) and multiparous (45.6%) women were included in this study.

**Table 1:** Maternal demographics

Maternal characteristics	*n* = 916	Mean ± SD, %
Age (years)	916	30.54 ± 5.0
Race		
Chinese	464	50.7
Indian	98	10.7
Malay	250	27.3
Other	104	11.4
Missing	0	0
Education		
Secondary school or under	11	1.2
High school	205	22.4
Junior college	365	39.8
University or above	332	36.2
Missing	3	0.3
Marital status		
Married	862	94.1
Single/divorced/widowed	54	5.9
Missing	0	0
Occupation		
White‐collar worker	731	79.8
Blue‐collar worker	175	19.1
Unemployed	10	1.1
Missing	0	0
Total monthly household income (S$)		
<3500	315	34.4
3500–5500	276	30.1
5501–8500	202	22.1
>8500	121	13.2
Missing	2	0.2
Parity		
Nulliparous	498	54.4
Multiparous	418	45.6
Missing	0	0

Maternal demographics – Categorical variables are expressed as %. Continuous variables are expressed as mean ± SD.

Lifestyle, BMI, blood pressure, uterine artery Doppler and sleep quality throughout pregnancy are presented in Table 2. A small percentage of women continued to drink coffee during pregnancy (21–33.4%). A smaller percentage of women remained actively drinking (0.8–2.5%) or smoking (1.8–2.6%) during pregnancy. The average BMI of the patients increased expectedly throughout pregnancy (from 24.1 at the first visit to 28.1 at the last). The average SBP/DBP (MAP) in the four visits were 108/66 (80) mmHg, 109/65 (79) mmHg, 110/66 (81) mmHg and 112/69 (83) mmHg respectively, showing an overall upward trend. Uterine artery RI progressively decreased from median of 0.8 during the first antenatal visit to 0.5 during the last visit. Uterine artery PI similarly decreased from a median of 1.8 during first visit to 0.6 during the last. Sleep quality score, as represented by PSQI, increased from a median of 6 at the first visit to 7 at the last visit. The median of individual aspects of sleep quality including sleep duration (7.0 hours), latency (15 minutes) and efficiency (87.5% in the first visit to 85.7% in the last) are also shown in Table 2. Based on the mean value, the overall sleep quality and sleep efficiency worsened, sleep duration shortened and sleep latency increased as pregnancy progressed.

**Table 2:** Maternal lifestyle, BMI, sleep quality, blood pressure measurements and uterine artery Dopplers throughout four antenatal visits

	1st visit (11–14 weeks)		2nd visit (18–22 weeks)		3rd visit (28–32 weeks)		4th visit (>34 weeks)	
	*n*	Value	*n*	Value	*n*	Value	*n*	Value
Lifestyle and BMI								
Coffee drinking (yes, %)	916	21.0	913	33.4	890	34.5	795	32.2
Smoking (yes, %)	916	2.6	913	2.8	890	1.8	795	1.8
Drinking alcohol (yes, %)	916	1.2	913	2.5	890	0.8	795	0.8
Exercise (yes, %)	916	8.4	913	13.5	890	12.5	795	12.8
BMI (kg/m^2^, mean ± SD)	911	24.1 ± 4.6	910	25.2 ± 4.5	889	27.1 ± 4.5	795	28.1 ± 4.8
BP								
SBP (mmHg, mean ± SD)	912	108.4 ± 11.2	911	108.5 ± 11.6	890	110.4 ± 11.1	797	112.4 ± 11.8
DBP (mmHg, mean ± SD)	912	65.7 ± 8.2	911	64.5 ± 8.0	890	65.5 ± 7.8	797	68.8 ± 8.7
MAP (mmHg, mean ± SD)	912	79.9 ± 8.5	911	79.1 ± 8.5	890	80.5 ± 8.1	797	83.3 ± 9.0
UA doppler								
UA_RI	896	0.8 (0.7–0.8)	866	0.6 (0.5–0.6)	815	0.5 (0.4–0.5)	695	0.5 (0.4–0.5)
UA_PI	896	1.8 (1.4–2.2)	866	1.0 (0.8–1.2)	815	0.7 (0.6–0.9)	695	0.6 (0.6–0.8)
Sleep								
PSQI	909	6.0 (4.0–8.0)	900	6.0 (4.0–8.0)	877	7.0 (5.0–9.0)	785	7.0 (5.0–10.0)
Latency (minute)	915	15.0 (10.0–30.0)	904	15.0 (10.0–30.0)	879	15.0 (10.0–30.0)	788	15.0 (10.0–30.0)
Duration (hour)	914	7.0 (6.0–8.0)	904	7.0 (6.0–8.0)	878	7.0 (6.0–8.0)	786	7.0 (5.0–8.0)
Efficiency (%)	912	87.5 (77.8–96.6	903	87.5 (76.9–96.9)	877	85.7 (75.0–94.4)	786	85.7 (71.4–95.0)
Sleep								
PSQI (mean ± SD)	909	6.6 ± 3.1	900	6.3 ± 3.2	877	7.2 ± 3.4	785	8.0 ± 3.3
Latency (minute, mean ± SD)	915	20.8 ± 31.6	904	20.2 ± 24.5	879	22.5 ± 23.4	788	25.7 ± 30.6
Duration (hour, mean ± SD)	914	7.0 ± 1.6	904	6.9 ± 1.8	878	6.6 ± 1.6	786	6.5 ± 1.7
Efficiency (% mean ± SD)	912	84.6 ± 14.9	903	84.6 ± 15.5	877	82.8 ± 15.8	786	81.8 ± 16.4

Continuous variables are expressed in mean ± SD if normally distributed and as median with interquartile range if not normally distributed. Categorical variables are expressed as %. Sleep was also expressed as mean ± SD.

During the first visit, SBP (*P* = 0.019), DBP (*P* = 0.023) and MAP (*P* = 0.014) were all significantly lower in women with longer duration of sleep (Table 3). Likewise, SBP (*P* = 0.016), DBP (*P* = 0.017) and MAP (*P* = 0.014) were significantly lower in women with a better sleep efficiency (Table 3). No significant difference in BP was found in women with different sleep qualities during the second and fourth visits (Table 3). DBP (*P* = 0.011) and MAP (*P* = 0.027) were significantly lower in subjects with better sleep efficiency during the third visit (Tables 3 and 4).

**Table 3:** Stepwise linear regression analysis of the correlation between sleep (log) and blood pressure in each antenatal visit

	SBP		DBP		MAP	
	B	P	B	P	B	P
1st visit (11–14 weeks)						
PSQI	1.784	0.204	1.636	0.124	1.603	0.137
Sleep latency (minute)	0.690	0.426	0.640	0.325	0.670	0.309
Sleep duration (hour)	−6.880	0.019*	−5.026	0.023*	−5.502	0.014*
Sleep efficiency (%)	−8.054	0.016*	−6.087	0.017*	−6.294	0.014*
2nd visit (18–22 weeks)						
PSQI	−0.186	0.892	−0.492	0.614	−0.390	0.697
Sleep latency (minute)	1.368	0.126	−0.406	0.524	0.727	0.265
Sleep duration (hour)	−4.976	0.087	−2.150	0.300	−3.092	0.145
Sleep efficiency (%)	−2.355	0.463	−0.965	0.673	−1.428	0.542
3rd visit (28–32 weeks)						
PSQI	0.347	0.808	1.351	0.189	1.128	0.275
Sleep latency (minute)	0.743	0.376	0.617	0.308	0.628	0.300
Sleep duration (hour)	−2.465	0.373	−2.921	0.143	−2.995	0.132
Sleep efficiency (%)	−2.392	0.462	−6.003	0.011*	−5.244	0.027*
4th visit (>34 weeks)						
PSQI	−3.004	0.148	−1.162	0.442	−1.541	0.329
Sleep latency (minute)	−0.694	0.473	−0.268	0.705	−0.497	0.498
Sleep duration (hour)	1.844	0.568	−0.982	0.764	−0.110	0.964
Sleep efficiency (%)	7.4312	0.058	2.955	0.307	3.628	0.223

Statistical analysis was done with adjustment of age, parity, BMI, lifestyle, race, marital status, occupation, education level and income.

* Statistical significance is defined as *P* < 0.05.

**Table 4:** Mixed model analysis of the correlation between sleep (log) and blood pressure

	SBP		DBP		MAP	
	B	P	B	P	B	P
PSQI	0.93	0.255	2.55	<0.001*	0.69	0.139
Sleep latency (minute)	0.45	0.282	0.68	0.018*	0.23	0.337
Sleep duration (hour)	−2.97	0.049*	−2.94	0.008*	0.46	0.359
Sleep efficiency (%)	−1.25	0.559	−3.38	0.008*	0.44	0.466

Statistical analysis was done with adjustment of all factors in Tables 1 and 2. Repeated covariance type: first order was used.

* Statistical significance is defined as *P* < 0.05.

When overall sleep and BP were assessed throughout the entire pregnancy, a lower PSQI score (*P* < 0.001), shorter sleep latency (*P* = 0.008) and better sleep efficiency (*P* = 0.008) were found to be correlated with lower DBP (Table 4). Longer sleep duration was associated with lower SBP (*P* = 0.049) and DBP (*P* = 0.008) (Table 4).

Assessment of the overall relationship between sleep and uterine artery Doppler throughout pregnancy showed that poorer sleep quality (higher PSQI, longer sleep latency, shorter sleep duration and worse sleep efficiency) were associated with higher uterine artery PI (Table 5). Patients with higher PSQI and shorter sleep duration were also found to have higher uterine artery RI (Table 5).

**Table 5:** Mixed model analysis of the correlation between sleep (log) and UA (log)

	UA RI		UA PI	
	B	P	B	P
PSQI	0.014	<0.001*	0.051	<0.001*
Sleep latency (minute)	0.005	0.057	0.014	0.017*
Sleep duration (hour)	−0.030	0.008*	−0.071	<0.001*
Sleep efficiency (%)	−0.014	0.177	−0.050	0.031*

Statistical analysis was done with adjustment of all factors in Tables 1 and 2. Repeated covariance type: first order was used.

* Statistical significance is defined as *P* < 0.05.

## Discussion

### Main findings

Our results showed that blood pressure (SBP and/or DBP and/or MAP) was lower in pregnant women with better sleep quality: shorter sleep latency, longer duration of sleep, better sleep efficiency and lower PSQI scores. Uterine artery PI was similarly lower in women with better sleep quality. The association between sleep and BP was the most significant in the first trimester as demonstrated by liner regression analysis.

### Strengths and limitations of study

Our study is the first large cohort study that has demonstrated a gradual change in sleep quality along with blood pressure changes throughout pregnancy, via which the association between sleep and BP was better scrutinised. As sleep pattern may gradually change over time, sleep quality in the first visit may not be representative of the overall sleep pattern throughout pregnancy. The association between sleep and BP was individually assessed at each visit, which accounts for the most direct association, if any. Our study is also the first study that has demonstrated a significant association between sleep and uterine artery Doppler, although the mechanism remains unclear. Lastly, the follow‐up rate of our study is very high, which makes the data more reliable.

Blood pressure varies throughout the day and is affected by activities immediately prior to blood pressure measurement. Our study only used a single point blood pressure during each visit for assessment. This may have led to some inaccuracy and error, as it may not be representative of the actual blood pressure. If possible, 24‐hour ambulatory pressure would be a better modality for the accurate assessment of blood pressure, although it is logistically much more difficult to execute. Presence of sleep disorders such as sleep apnoea and subjects’ psychiatric history, e.g. a history of depression, were not explored in our study. These factors can potentially affect sleep quality and blood pressure and it would be ideal if these factors could be adjusted in the analysis.

PCOS and insulin resistance are known comorbidities that can potentially affect sleep quality and blood pressure. Studies have shown that people with PCOS had a higher incidence of snoring, obstructive sleep apnoea and poorer sleep quality.^25^ Insulin resistance is a stronger risk factor than is body mass index or testosterone for sleep‐disordered breathing in PCOS women.^26^ PCOS patients with hyperinsulinaemia were also found to have higher BP.^27^ However, our study did not have information on a background of PCOS or insulin resistance of our subjects and we were therefore unable to control for them during statistical analysis.

### Interpretation

Sleep latency, duration and efficiency were important aspects of sleep with a significant association with BP. A previous cohort study by William et al.^15^ found that mean first and second trimester SBP and DBP values were similar among women reporting to be short sleepers (≤6 hours) versus women reporting to sleep >9 hours. However, both short and long sleep duration in early pregnancy were associated with increased mean third trimester SBP and DBP compared with women who sleep for 9 hours in their study.^15^ Their findings were in line with some previous studies done in the non‐pregnant population.^1^, ^2^, ^3^, ^4^ Okada et al.^17^ found that poor sleep quality (defined as PSQI ≥ 6) resulted in a greater increase in blood pressure in the third trimester from the first trimester compared with good sleepers (defined as PSQI ≤5).

The analysis in our study differed from these previous studies. Sleep duration, sleep efficiency, latency and PSQI were considered as continuous variables in mixed model and linear regression analysis instead of as categorical variables as in those studies. We believe that the association analysis using continuous variables offers a more comprehensive comparison. Although sleep in early pregnancy may affect BP in late pregnancy, it is more likely that BP is continuously influenced by the dynamic change of sleep quality throughout pregnancy and therefore the association is better investigated with collective data reflecting the entire pregnancy.

The mechanism linking sleep and blood pressure is very complex. One possible mechanism is increased oxidative stress and reduced nitric oxide (NO) in poor sleepers. NO is an important mediator in vascular function causing dilation of the vessels and thus resulting in lower BP. In the case of obstructive sleep apnoea, nocturnal hypoxaemia induces oxidative stress, which triggers an inflammatory response and a reduction in NO.^28^, ^29^ Another possible mechanism is through the sympathetic pathway and the hypothalamic‐pituitary‐adrenal axis. In some experimental studies, increased secretion of catecholamines, altered sympathovagal balance and disruption in cortisol levels were postulated to link poor sleep with elevated blood pressure.^30^, ^31^, ^32^, ^33^ A few interesting studies showed that an increase in pro‐inflammatory cytokines such as interleukin‐1 and tumour necrosis factor‐alpha alter sleep pattern and may potentially cause elevated blood pressure.^34^, ^35^, ^36^ However, the exact underlying mechanism remains to be elucidated.

Study on the association between sleep quality and uterine artery blood flow is scarce in the literature. Whereas Robertson et al.^22^ found no significant influence of sleep on uterine artery PI, our study demonstrated that uterine artery PI and RI were significantly lower in women with better sleep. One postulation is that poor sleep may lead to suboptimal trophoblastic invasion of myometrium and consequently higher blood flow resistance. In other words, higher blood pressure in people with poorer sleep can potentially be caused by placental dysfunction. Another possible explanation is that sleep is associated with worse uterine artery Doppler and higher blood pressure via a common mechanism such as reduced NO which leads to vasoconstriction.

The association between sleep disturbances and higher blood pressure demands that clinicians and researchers recognise the importance of sleep during pregnancy and explore ways to improve sleep antenatally. Most obstetric units do not routine screen for sleep disruption. A short questionnaire on sleep quality can be incorporated into routine obstetric care. Early identification of patients at risk of sleep disturbances and intervention at an early stage may benefit pregnancy outcome. Studies on potential interventions in improving sleep in pregnancy are rather limited. Stremler et al. conducted a randomised controlled trial on the effect of behavioural‐educational intervention in improving sleep in postpartum women. Although no significance was found in the primary outcome of nocturnal sleep, it is still worth exploring intervention during early pregnancy instead of postpartum, when sleep is significantly interfered by breastfeeding. Another interesting study recently reported a significant improvement of sleep in terms of longer sleep duration and less sleep disruption with a home‐based cognitive‐behavioural training programme during late pregnancy.^37^ Further studies on possible interventions antenatally to improve sleep and potentially lower blood pressure would be valuable.

## Conclusion

Our cohort study is among the first to show an association between sleep and blood pressure throughout pregnancy in the population without a pre‐existing hypertensive disorder. The most significant association was found in the first trimester. Uterine artery Doppler was worse in poorer sleepers, suggesting a potential association between sleep and placental function which may affect blood pressure. Future studies should focus on interventions to improve sleep antenatally in an effort to optimise blood pressure and pregnancy outcome.

### Disclosure of interests

The authors report grants from the Singapore National Medical Research Council (NMRC) Collaborative Centre Grant during the conduct of the study. There are no other conflicts of interest to declare. Completed disclosure of interests forms are available to view online as supporting information.

### Contribution to authorship

YT contributed to the design of the study, data analysis and interpretation, manuscript drafting and review. JZ, FD and KHT contributed to the design of the study, data analysis, data interpretation and review of manuscript to be published. KHT, NSR, ST and BC contributed to the conception and design of the study, and review of manuscript to be published.

### Details of ethics approval

A written informed consent was obtained from all participants and the study was approved by the SingHealth Centralised Institutional Review Board Ethics Committee, Singapore (CIRB Ref No. 2010/214/D) on 4 June 2010.

### Funding

This study is supported by Singapore National Medical Research Council (NMRC) Collaborative Centre Grant grant − Integrated Platform for Research in Advancing Metabolic Health Outcomes of Women and Children, IPRAMHO/CGAug16C008) and Neonatal & Obstetric Risk Assessment (NORA) prospective cohort study (Project Grant number NMRC/PPG/KKH/2010).

## Supporting information

Supplementary Material

Supplementary Material

Supplementary Material

Supplementary Material

Supplementary Material

Supplementary Material

Supplementary Material

## Data Availability

The data that support the findings of this study are available on request from the corresponding author. The data are not publicly available due to privacy or ethical restrictions.
